# Willingness to Share Data From Wearable Health and Activity Trackers: Analysis of the 2019 Health Information National Trends Survey Data

**DOI:** 10.2196/29190

**Published:** 2021-12-13

**Authors:** Camella J Rising, Anna Gaysynsky, Kelly D Blake, Roxanne E Jensen, April Oh

**Affiliations:** 1 Behavioral Research Program Division of Cancer Control and Population Sciences US National Cancer Institute Rockville, MD United States; 2 Health Care Delivery Research Program Division of Cancer Control and Population Sciences US National Cancer Institute Rockville, MD United States; 3 Implementation Science, Office of the Director Division of Cancer Control and Population Sciences US National Cancer Institute Rockville, MD United States

**Keywords:** mobile health, population health, health communication, survey methodology, mobile apps, devices, online social networking, mobile phone

## Abstract

**Background:**

Sharing data from wearable health and activity trackers (wearables) with others may improve the health and behavioral outcomes of wearable users by generating social support and improving their ability to manage their health. Investigating individual factors that influence US adults’ willingness to share wearable data with different types of individuals may provide insights about the population subgroups that are most or least likely to benefit from wearable interventions. Specifically, it is necessary to identify digital health behaviors potentially associated with willingness to share wearable data given that the use of and engagement with various technologies may broadly influence web-based health information–sharing behaviors.

**Objective:**

This study aims to identify sociodemographic, health, and digital health behavior correlates of US adults’ willingness to share wearable data with health care providers and family or friends.

**Methods:**

Data for the analytic sample (N=1300) were obtained from the 2019 Health Information National Trends Survey of the National Cancer Institute. Digital health behavior measures included frequency of wearable device use, use of smartphones or tablets to help communicate with providers, use of social networking sites to share health information, and participation in a web-based health community. Multivariable logistic regression analysis of weighted data examined the associations between digital health behaviors and willingness to share wearable device data, controlling for sociodemographics and health-related characteristics.

**Results:**

Most US adults reported willingness to share wearable data with providers (81.86%) and with family or friends (69.51%). Those who reported higher health self-efficacy (odds ratio [OR] 1.97, 95% CI 1.11-3.51), higher level of trust in providers as a source of health information (OR 1.98, 95% CI 1.12-3.49), and higher level of physical activity (OR 2.00, 95% CI 1.21-3.31) had greater odds of willingness to share data with providers. In addition, those with a higher frequency of wearable use (OR 2.15, 95% CI 1.35-3.43) and those who reported use of smartphones or tablets to help communicate with providers (OR 1.99, 95% CI 1.09-3.63) had greater odds of willingness to share data with providers. Only higher level of physical activity was associated with greater odds of willingness to share wearable data with family or friends (OR 1.70, 95% CI 1.02-2.84). Sociodemographic factors were not significantly associated with willingness to share wearable data.

**Conclusions:**

The findings of this study suggest that, among US adult wearable users, behavior-related factors, rather than sociodemographic characteristics, are key drivers of willingness to share health information obtained from wearables with others. Moreover, behavioral correlates of willingness to share wearable data are unique to the type of recipient (ie, providers vs family or friends). Future studies could use these findings to inform the development of interventions that aim to improve the use of patient-generated data from wearable devices in health care settings.

## Introduction

### Background

In 2019, nearly one-quarter of US adults reported using wearable health and activity trackers (wearables) [1-3], which is approximately twice the reported use in 2015 [4]. Wearables are mobile health (mHealth) technologies worn on the body that can detect, record, and report information about behaviors (eg, step count and dietary intake) and health indicators (eg, heart rate and calories burned) [5]. Wearables may improve health and behavioral outcomes, such as physical activity participation [1,6] and weight status [7], by prompting users to set health goals, providing automated personalized feedback about health and activity data, motivating healthy habit formation, and encouraging social data sharing and competition [6-9].

Connected devices, such as mHealth apps on smartphones or computer tablets, enable users to share health information from wearables with others, such as health care providers and family or friends. Sharing wearable data may improve the health and behavioral outcomes of users by generating social support and improving their ability to manage their health [9-12], increasing patient–provider engagement, and facilitating individualized counseling and clinical decision making [12-15]. Studies also suggest that wearable data sharing among behavioral intervention participants may increase intervention effectiveness [16].

When used as a health communication tool, the potential of wearable technologies to improve health may not be fully realized without an understanding of the willingness to share wearable data, particularly with providers. Reasons for not sharing wearable data with providers include lack of awareness of the social sharing features of wearables, uncertainty about the relevance or usefulness of the data to providers, low expectation of supportive feedback, and concerns about privacy or control over data shared from the device [12-14]. Past studies of patient data sharing from various mHealth technologies also suggest that willingness to share wearable data may vary by individual characteristics, such as sociodemographics (eg, sex, age, race, or ethnicity), health-related factors (eg, weight status or having a chronic condition), and trust [17-19]. However, the correlates of willingness to share data from wearable health and activity trackers, specifically, need further examination, as the characteristics of mHealth users vary by type of device used for health and behavioral tracking [20].

Following evidence that the use of and engagement with digital health can influence health and communication behavior [21-23], digital health behaviors may be additional factors associated with the willingness to share wearable data. For example, individuals who use mHealth technologies such as smartphones or tablets to help communicate with providers may have greater technology self-efficacy [21], which may influence their willingness to share health information from wearable devices. Frequency of wearable use, one aspect of engagement with mHealth technologies for health and behavioral tracking [22], could also be a factor associated with willingness to share data from these devices. Moreover, other digital health behaviors, such as sharing health information on social networking sites (SNSs; eg, Facebook) or within web-based health communities (eg, online cancer support groups), may be associated with willingness to share wearable data if social sharing of health information across digital media is broadly perceived as useful or beneficial [9,11,23]. However, to the best of our knowledge, such relationships between digital health behaviors and willingness to share data from wearables have not been examined in a nationally representative sample of US adults.

A better understanding of the factors that influence willingness to share data from wearables could have implications for the use of patient-generated data in clinical practice [24], particularly given the growing number of interventions that use wearable devices to track health and activity [25] and recent calls for integration and use of these data in interventions [26,27]. Identifying the sociodemographic, health, and behavioral correlates of willingness to share data from wearables could provide insights on the population subgroups that are most or least likely to engage with, and benefit from, wearable interventions or multicomponent behavioral interventions that involve wearable use. In addition, exploring the correlates of willingness to share wearable data with providers, as well as with family or friends, may identify different drivers of willingness to share data with different types of recipients.

### Objectives

This study has 2 primary aims to address gaps in the literature. The first aim is to describe the sociodemographic and health-related correlates of the reported willingness of wearable users to share data with health care providers and with family or friends. The second aim is to investigate the relationship between different digital health behaviors (ie, use of smartphones or tablets to help communicate with providers, frequency of wearable use, sharing health information on SNSs, and participation in a web-based health community) and the willingness of users to share wearable data with health care providers and with family or friends.

## Methods

### Sample Population

Data from the 2019 Health Information National Trends Survey (HINTS) of the National Cancer Institute were analyzed. HINTS is a nationally representative, probability-based cross-sectional survey. Self-administered questionnaires were completed by adult, civilian, noninstitutionalized individuals (N=5438) between January and April 2019 (Multimedia Appendix 1). Respondents completed mailed paper questionnaires (paper-only group) or completed the questionnaire on the web as part of a push-to-web pilot study. Individuals participating in the web pilot were randomly assigned to a web-option group (choice of responding by paper or web) or a web-bonus group (choice of responding by paper or web, with a US $10 bonus incentive for responding via web). There were no significant differences in response rates for the paper-only group (30.2%), the web-option group (29.6%), and the web-bonus group (31.5%). Additional information about HINTS data, resources, and methodology has been described elsewhere [28], and information specific to HINTS 5 Cycle 3 (2019) can be found in publicly available methods reports [29].

To be included in the analytic sample, respondents had to report the use of a wearable device to track their health or activity. Thus, respondents were included if they selected “yes” (vs “no”) in response to the item “In the past 12 months, have you used an electronic wearable device to monitor or track your health or activity? For example, a Fitbit, Apple Watch, or Garmin Vivofit*.*” In addition, respondents must have reported data for the outcome variables of interest, as described in the following sections.

### Measures

#### Willingness to Share Wearable Data

Willingness to share wearable data with providers was measured with the item “Would you be willing to share health data from your wearable device with your health care provider?” (“yes” or “no”). Willingness to share wearable data with family or friends was measured by asking respondents, “Would you be willing to share health data from your wearable device with your family or friends?” (“yes” or “no”).

#### Digital Health Behaviors

To evaluate web-based health information sharing, the reported use of SNSs to share health information and participate in a web-based health community were examined. The use of SNSs was measured with the item “In the past 12 months, have you used the Internet for any of the following reasons? To share health information on social networking sites, such as Facebook or Twitter” (“yes” or “no”). To measure participation in a web-based health community, respondents were asked: “In the past 12 months, have you used the Internet for any of the following reasons? To participate in an online forum or support group for people with a similar health or medical issue” (“yes” or “no”).

The use of mHealth technologies to help communicate with providers was measured with the item “Has your tablet or smartphone helped you in discussions with your health care provider?” (“yes” or “no”). Frequency of wearable use was evaluated by asking respondents: “In the past month, how often did you use a wearable device to track your health?” Responses were dichotomized into higher frequency use (“almost every day” or “every day”) and lower frequency use (“1-2 times per week,” “less than once per week,” or “did not use a wearable device in the past month”).

#### Health-Related Characteristics

Health-related correlates included perceived health status, health self-efficacy, BMI, multimorbidity, and level of physical activity. Perceived health status was measured with the item “In general, would you say your health is...?” Responses were dichotomized into good health (“excellent,” “very good,” or “good”) and “fair” or “poor” health. Health self-efficacy was measured with the item “Overall, how confident are you about your ability to take good care of your health?” Responses were dichotomized into higher health self-efficacy (“very confident” or “completely confident”) and lower health self-efficacy (“somewhat confident,” “a little confident,” or “not confident at all”). Self-reported height and weight were used to calculate and classify BMI [30]; underweight respondents were excluded from analysis due to low frequency of BMI indicative of underweight (BMI<18.5) among wearable users (n=13). A composite multimorbidity variable (0 conditions, 1 condition, or ≥2 conditions) combined data from items that assessed history of chronic conditions (“yes” or “no”), including diabetes, heart disease, lung disease, depression or anxiety, and any cancer except nonmelanoma skin cancer. On the basis of the Physical Activity Guidelines for Americans for minutes per week of moderate-intensity physical activity [31], level of physical activity was assessed with a discrete numerical response to the item “On the days that you do any physical activity or exercise of at least moderate intensity, how long do you typically do these activities?” Responses were dichotomized as higher level of physical activity (≥150 minutes per week) versus lower level of physical activity (<150 minutes per week).

Additional health-related measures included having a regular health care provider, trust in health information from a physician, and trust in health information from family or friends. Having a regular health care provider (“yes” or “no”) was measured with the item “Not including psychiatrists and other mental health professionals, is there a particular doctor, nurse, or other health professional that you see most often?” Trust in health information from a physician was evaluated with the item “In general, how much would you trust information about health or medical topics from each of the following? A doctor.” Trust in health information from family or friends was evaluated with the item “In general, how much would you trust information about health or medical topics from each of the following? Family or friends.” Response options for both trust items were dichotomized as higher trust (“a lot”) versus lower trust (“some,” “a little,” or “not at all”).

#### Sociodemographic Characteristics

Sociodemographic variables included sex (women and men), age (18-34, 35-49, 50-64, and ≥65 years), race (White, Black, and other races, which combined low-frequency responses for American Indian or Alaska Native, Asian Indian, Chinese, Filipino, Japanese, Korean, Vietnamese, other Asian, Native Hawaiian, Guamanian or Chamorro, Samoan, and other Pacific Islander), ethnicity (Hispanic and non-Hispanic), education (high school graduate or less; technical, vocational, or some college; and college graduate or postgraduate), annual household income in US dollars (<US $35,000, US $35,000-$49,999, US $50,000-$74,999, US $75,000-$99,999, and ≥US $100,000), and geographic area (urban vs rural [32]). Due to the relatively high proportion of missing data in the annual household income measure, an imputed variable provided in the data set was used to avoid losing respondents in the analytic sample.

### Statistical Analysis

Frequencies, weighted percentages, and chi-square statistics were calculated to describe the distribution of US adults who reported using a wearable device to track health or activity. Binomial logistic regression analysis was conducted to examine correlations between individual characteristics (sociodemographic, health-related, and digital health behavior variables) and willingness to share wearable data with health care providers, and correlations between individual characteristics and willingness to share wearable data with family or friends. In total, 2 regression models were constructed.

For the model predicting willingness to share wearable data with providers, all sociodemographic and health-related variables (excluding trust in health information from family or friends) were entered first to address the first aim of this study. Because digital health behaviors were factors of particular interest, the second aim was addressed by adding digital health behavior variables stepwise in the following order: frequency of wearable use, use of mHealth technologies to help communicate with providers, use of SNSs to share health information, and participation in a web-based health community. The order in which variables were added to the model was based on the extent of supporting literature [21-23] that suggests a potential association between the respective digital health behavior and willingness to share wearable data, such that variables with a greater evidence base were added to the model first. Pseudo *R^2^* was examined after adding each digital health behavior variable to the model to determine how much variability could be explained by each of these key predictor variables. For the model predicting willingness to share wearable data with family or friends, covariates were entered using a similar stepwise approach; however, variables pertaining to interactions with providers were excluded (ie, use of mHealth technologies to help communicate with providers, having a regular health care provider, and trust in health information from a physician), whereas trust in health information from family or friends was added.

Statistical analysis was conducted using SAS (version 9.4; SAS Institute). Complete-case analysis with listwise deletion was used for the regression models. Group differences by survey modality (paper-only, web-option, and web-bonus) for the outcome variables of interest were assessed using the jackknife replication variance estimation method, applying a final sample weight and replicate weights created using the Rizzo method [33]. Because group differences were not significant in our analysis of outcomes by modality, the full-sample weight was applied to calculate population estimates for the combined sample without controlling for group differences by survey modality. Replicate weights were also used to compute SEs of estimates using the jackknife replication method for the combined sample without controlling for group differences by survey modality.

## Results

### Sample Population

The analytic sample comprised 1300 wearable users. Analysis of weighted data showed that women (55.03%) and men (44.97%) each constituted approximately half of the sample. The majority were under 50 years of age (64.1%), urban residents (88.07%), non-Hispanic White (64.69%), and reported having an education beyond high school (85%). Individuals with annual household income under US $75,000 comprised 44.02% of the sample, with the remaining 55.98% having an annual household income of US $75,000 or more (Table 1). The characteristics of the HINTS analytic sample can be referenced alongside the characteristics of the analytic sample of wearable users in Multimedia Appendix 2. Similar to other health-based surveys, HINTS respondents tend to be female, older, non-Hispanic White, urban-dwelling, and more educated, and have a higher annual household income than the general population [28].

Most individuals reported having a good health status (89.65%), higher health self-efficacy (77.15%), and a regular health care provider (64.73%). More individuals reported higher (vs lower) trust in health information from a physician (75.55%) and lower (vs higher) trust in health information from family or friends (90.89%). A majority of individuals had a BMI≥24.9 (69.44%), and just over half reported having one or more chronic conditions (53.71%). Approximately half of the individuals reported a higher (48.54%) versus lower (51.46%) level of physical activity.

Most individuals included in the analytic sample reported using their wearables “every day” or “almost every day” (72%). They were relatively evenly divided on the use of other mHealth technologies (eg, smartphones and tablets) to help communicate with providers (47.93% “yes” vs 52.07% “no”). A minority of individuals reported sharing health information on SNSs (19.54%) or participating in a web-based health community (11.95%).

**Table 1:** Weighted, unadjusted population estimates for sociodemographic and health-related characteristics of wearable users willing to share data with providers and with family or friends, HINTS 2019^a^ (N=1300).

Characteristics		Users, n (weighted %, SE)		
		Wearable users (N=1300)	Willing to share data with providers (n=1033^b^)	Willing to share data with family or friends (n=853^c^)
**Sex**				
	Men	486 (44.97, 2.08)	387 (44.44, 2.24)	310 (44.68, 2.62)
	Women	787 (55.03, 2.08)	630 (55.56, 2.24)	527 (55.32, 2.62)
**Age (years)**				
	18-34	272 (33.62, 2.08)	234 (36.42, 2.46)	211 (36.87, 3.03)
	35-49	339 (30.48, 1.85)	265 (29.82, 2.32)	237 (30.62, 2.49)
	50-64	411 (26.37, 1.88)	315 (24.81, 2.11)	245 (23.91, 2.3)
	≥65	257 (9.53, 0.81)	205 (8.95, 0.87)	152 (8.6, 0.97)
**Race and ethnicity**				
	White, non-Hispanic	788 (64.69, 1.83)	645 (66.21, 2.03)	533 (66.04, 2.6)
	Black, non-Hispanic	141 (8.86, 1.19)	114 (9.26, 1.54)	96 (9.82, 1.92)
	Hispanic	169 (17.66, 1.64)	129 (16.29, 1.81)	103 (15.93, 1.86)
	Other race or ethnicity	106 (8.79, 1.18)	78 (8.24, 1.32)	70 (8.21, 1.47)
**Education**				
	College graduate or postgraduate	796 (42.55, 1.97)	647 (43.98, 2.41)	541 (43.69, 2.63)
	Technical, vocational, or some college	344 (42.45, 2.38)	276 (43.38, 2.84)	220 (41.83, 3.1)
	High school graduate or less	130 (15, 2.07)	90 (12.64, 2.05)	76 (14.48, 2.57)
**Annual household income (US $)**				
	≥100,000	526 (38.26, 2.25)	424 (38.41, 2.17)	357 (39.39, 2.76)
	75,000-99,999	211 (17.72, 1.56)	175 (19.18, 1.94)	138 (17.5, 2.1)
	50,000-74,999	219 (16.18, 1.58)	176 (16.02, 1.74)	142 (16.33, 2.03)
	35,000-49,999	142 (13.8, 1.81)	105 (12.6, 2.1)	96 (13.38, 2.22)
	<35,000	192 (14.04, 1.77)	145 (13.79, 1.82)	112 (13.4, 1.85)
**Geographic area**				
	Urban	1196 (88.07, 1.91)	951 (88.25, 2.26)	780 (86.07, 2.65)
	Rural	104 (11.93, 1.91)	82 (11.75, 2.26)	73 (13.93, 2.65)
**Perceived health status**				
	Poor or fair	113 (10.35, 1.65)	85 (9.67, 1.85)	51 (7.42, 2.1)
	Good	1174 (89.65, 1.65)	938 (90.33, 1.85)	793 (92.58^d^, 2.1)
**Health self-efficacy**				
	Lower	278 (22.85, 1.97)	209 (20.32, 2.17)	161 (18.54, 2.33)
	Higher	1006 (77.15, 1.97)	813 (79.68, 2.17)	683 (81.46^e^, 2.33)
**Regular health care provider**				
	No	379 (35.27, 2.28)	288 (33.6, 2.74)	Not examined^f^
	Yes	904 (64.73, 2.28)	735 (66.4, 2.74)	Not examined^f^
**Trust health information from physician**				
	Lower	321 (24.45, 1.9)	226 (20.96, 2.24)	Not examined^f^
	Higher	955 (75.55, 1.9)	794 (79.04^g^, 2.24)	Not examined^f^
**Trust health information from family or friends**				
	Lower	1167 (90.89, 1.64)	Not examined^h^	772 (89.55, 2.42)
	Higher	89 (9.11, 1.64)	Not examined^h^	66 (10.45, 2.42)
**BMI**				
	18.5-24.9 (normal)	389 (30.56, 1.92)	313 (30.28, 2.34)	263 (30.09, 2.39)
	25-29.9 (overweight)	472 (38.87, 2.44)	374 (39.45, 2.93)	321 (40.46, 3.1)
	≥30 (obese)	395 (30.57, 2.15)	318 (30.27, 2.41)	244 (29.45, 2.78)
**Multimorbidity**				
	0 conditions	545 (46.29, 2.3)	429 (46.04, 2.83)	368 (47.62, 3.02)
	1 condition	398 (31.56, 2.31)	320 (31.46, 2.6)	271 (33.35, 3.02)
	≥2 conditions	326 (22.15, 2.02)	263 (22.5, 2.55)	193 (19.03, 2.38)
**Level of physical activity**				
	Lower	674 (51.46, 2.18)	521 (47.97, 2.53)	430 (47.06, 2.81)
	Higher	595 (48.54, 2.18)	490 (52.03^i^, 2.53)	408 (52.94^j^, 2.81)

^a^HINTS 2019: Health Information National Trends Survey 5, Cycle 3.

^b^A total of 18 wearable users had missing data for willingness to share wearable data with providers, therefore the denominator for weighted percentages in this column is 1282.

^c^A total of 22 wearable users had missing data for willingness to share wearable data with family or friends, therefore the denominator for weighted percentages in this column is 1278.

^d^*χ*^2^_1_=5.9; *P*=.02.

^e^*χ*^2^_1_=7.2; *P*=.01.

^f^Provider-specific variables were not examined in the model predicting willingness to share wearable data with family or friends (see section *Statistical Analysis*).

^g^*χ*^2^_1_=8.0; *P*=.007.

^h^Family or friend-specific variables were not examined in the model predicting willingness to share wearable data with providers (see section *Statistical Analysis*).

^i^*χ*^2^_1_=12.5; *P*<.001.

^j^*χ*^2^_1_=7.6; *P*=.008.

### Willingness to Share Wearable Data

A small number of wearable users had missing data regarding willingness to share wearable data with providers (n=18) and willingness to share wearable data with family or friends (n=22); therefore, the analytic sample comprised 1282 respondents for willingness to share wearable data with providers and 1278 respondents for willingness to share wearable data with family or friends. A majority of individuals reported that they would be willing to share wearable data with health care providers (81.86%) and with family or friends (69.1%). In the bivariate analyses, willingness to share wearable data with providers was significantly associated with trust in health information from a physician and level of physical activity. Willingness to share wearable data with family or friends was significantly associated with perceived health status, health self-efficacy, and level of physical activity (Table 1).

Willingness to share wearable data with providers was also significantly associated with each of the 4 measured digital health behaviors: frequency of wearable use, use of mHealth technologies to help communicate with providers, use of SNSs to share health information, and participation in a web-based health community. Only the use of SNSs to share health information was significantly correlated with reported willingness to share wearable data with family or friends (Table 2).

Regression analysis showed that individuals who reported higher (vs lower) health self-efficacy (odds ratio [OR] 1.97, 95% CI 1.11-3.51), higher (vs lower) trust in health information from a physician (OR 1.98, 95% CI 1.12-3.49), and higher (vs lower) levels of physical activity (OR 2.00, 95% CI 1.21-3.31) had significantly greater odds of reported willingness to share wearable data with providers. Among the digital health behaviors, higher (vs lower) frequency of wearable use (OR 2.15, 95% CI 1.35-3.43) and use of mHealth technologies to help communicate with providers (OR 1.99, 95% CI 1.09-3.63) were significantly associated with willingness to share wearable data with providers (Table 3). On the basis of pseudo *R^2^* values, the model fit improved with the addition of each digital health behavior variable.

In the regression analysis, only individuals who reported higher (vs lower) levels of physical activity had higher odds of reported willingness to share wearable data with family or friends (OR 1.70, 95% CI 1.02-2.84; *P*=.04). Of the 3 digital health behaviors included in the model, none were significantly associated with willingness to share wearable data with family or friends (data not shown). As in the first model, the model fit improved with the addition of each digital health behavior variable.

**Table 2:** Weighted, unadjusted population estimates for digital health behaviors of wearable users willing to share data with providers and with family or friends, HINTS 2019^a^ (N=1300).

Characteristic			Users, n (weighted %, SE)				
			Wearable users (N=1300)		Willing to share data with providers (n=1033^b^)		Willing to share data with family or friends (n=853^c^)
**Frequency of wearable use**							
	Lower	396 (28, 1.69)		295 (25.84, 2.02)		237 (26.52, 2.42)	
	Higher	888 (72, 1.69)		735 (74.16^d^, 2.02)		615 (73.48, 2.42)	
**Use of mHealth^e^ technologies to help communicate with providers**							
	No	635 (52.07, 2.18)		482 (47.98, 2.68)		Not examined^f^	
	Yes	617 (47.93, 2.18)		521 (52.02^g^, 2.68)		Not examined^f^	
**Use social networking sites to share health information**							
	No	1056 (80.46, 1.75)		830 (78.51, 2.24)		670 (77.57, 2.35)	
	Yes	229 (19.54, 1.75)		192 (21.49^h^, 2.24)		174 (22.43^i^, 2.35)	
**Participating in an online health community**							
	No	1143 (88.05, 1.58)		901 (86.52, 2.08)		737 (86.7, 2.21)	
	Yes	145 (11.95, 1.58)		122 (13.48^j^, 2.08)		108 (13.3, 2.21)	

^a^HINTS 2019: Health Information National Trends Survey 5, Cycle 3.

^b^A total of 18 wearable users had missing data for willingness to share wearable data with providers, therefore the denominator for weighted percentages in this column is 1282.

^c^A total of 22 wearable users had missing data for willingness to share wearable data with family or friends, therefore the denominator for weighted percentages in this column is 1278.

^d^*χ*^2^_1_=4.27; *P*=.04.

^e^mHealth: mobile health.

^f^Provider-specific variables were not examined in the model predicting willingness to share wearable data with family or friends.

^g^*χ*^2^_1_=11.13; *P*=.002.

^h^*χ*^2^_1_=5.55; *P*=.02.

^i^*χ*^2^_1_=5.56; *P*=.02.

^j^*χ*^2^_1_=5.67; *P*=.02.

**Table 3:** Correlates of willingness to share wearable data with providers, weighted, fully adjusted binomial logistic regression model, HINTS 2019^a^ (n=1070).

Characteristic		OR^b^ (95% CI)	*P* value
**Sex**			
	Men	Reference	Reference
	Women	1.13 (0.68-1.89)	.63
**Age (years)**			
	18-34	Reference	Reference
	35-49	0.49 (0.25-0.98)	.05
	50-64	0.51 (0.24-1.09)	.08
	≥65	0.45 (0.17-1.22)	.11
**Race and ethnicity**			
	White, non-Hispanic	Reference	Reference
	Black, non-Hispanic	1.26 (0.50-3.18)	.62
	Hispanic	0.53 (0.25-1.11)	.09
	Other race or ethnicity	0.54 (0.20-1.49)	.23
**Education**			
	College graduate or postgraduate	Reference	Reference
	Technical, vocational, or similar college	1.05 (0.58-1.91)	.87
	High school graduate or less	0.73 (0.34-1.58)	.42
**Annual household income (US $)**			
	≥100,000	Reference	Reference
	75,000-99,999	1.69 (0.76-3.76)	.20
	50,000-74,999	1.38 (0.72-2.64)	.32
	35,000-49,999	0.86 (0.37-2.01)	.72
	<35,000	1.61 (0.64-4.06)	.30
**Geographic area**			
	Urban	Reference	Reference
	Rural	0.56 (0.25-1.22)	.14
**Perceived health status**			
	Poor or fair	Reference	Reference
	Good	0.96 (0.33-2.76)	.94
**Health self-efficacy**			
	Lower	Reference	Reference
	Higher	1.97 (1.11-3.51)	.02
**Regular health care provider**			
	No	Reference	Reference
	Yes	1.40 (0.75-2.61)	.28
**Trust in health information from a physician**			
	Lower	Reference	Reference
	Higher	1.98 (1.12-3.49)	.02
**BMI**			
	18.5-24.9 (normal)	Reference	Reference
	25-29.9 (overweight)	1.00 (0.48-2.08)	.99
	≥30 (obese)	0.97 (0.46-2.05)	.93
**Multimorbidity**			
	0 conditions	Reference	Reference
	1 condition	0.98 (0.53-1.83)	.94
	≥2 conditions	1.16 (0.49-2.76)	.74
**Level of physical activity**			
	Lower	Reference	Reference
	Higher	2.00 (1.21-3.31)	.008
**Frequency of wearable use**			
	Lower	Reference	Reference
	Higher	2.15 (1.35-3.43)	.002
**Use of mHealth^c^ technologies to help communicate with providers**			
	No	Reference	Reference
	Yes	1.99 (1.09-3.63)	.03
**Use of SNSs^d^ to share health information**			
	No	Reference	Reference
	Yes	1.50 (0.72-3.12)	.27
**Participation in a web-based health community**			
	No	Reference	Reference
	Yes	1.64 (0.65-4.15)	.29

^a^HINTS 2019: Health Information National Trends Survey 5, Cycle 3.

^b^OR: odds ratio.

^c^mHealth: mobile health.

^d^SNS: social networking site.

## Discussion

### Principal Findings

The purpose of this study was to describe the willingness to share health information collected on wearable health and activity trackers with health care providers and family or friends in a nationally representative sample of US adult wearable users. The findings of this study suggest that most individuals who used wearables were willing to share data generated from these devices with providers (approximately 80%), as well as with family or friends (approximately 70%); however, willingness to share this information varied with behavior-related factors. Health self-efficacy, trust in providers as an information source, frequency of wearable use, use of other mHealth technologies to help communicate with providers, and being physically active appeared to be key factors that influenced willingness to share wearable data with providers. Being physically active also appeared to play an important role in willingness to share data from wearables with family or friends, whereas other factors such as sociodemographics, health-related characteristics, and digital health behaviors played a less prominent role.

These findings contribute to the literature by identifying individual characteristics associated with willingness to share data from wearable health and activity trackers in the adult population and distinguishing the correlates of willingness to share on the basis of the recipient of the data. Interestingly, our study revealed no differential willingness to share according to the sociodemographic characteristics of wearable users. Although the HINTS response rate was relatively low for all survey modalities (approximately 30%), differences by survey modality group (paper, web, and web-bonus) were not significant for the response rate and for the outcome variables of interest.

### Willingness to Share Wearable Data With Health Care Providers

The findings of this study suggest that willingness to exchange health- and activity-related information with providers via mHealth technologies may be increasing. For example, in 2013, approximately 50% of US adults who used smartphones, tablets, or other mobile devices reported that they would be “somewhat” or “very” willing to use these technologies to exchange health information about lifestyle behaviors with a provider [18]. Similar to the results of this study, Hyde et al [2] found that approximately 76% of adults reported willingness to share data from wearable health and activity monitors or fitness trackers with providers.

In contrast to prior studies examining willingness or sharing of data from mHealth technologies, factors such as sex, age, weight status [17], race and ethnicity [17,34], income [18,19], and education [18] were not significantly associated with willingness to share wearable data with providers. In addition, having a regular health care provider and having a chronic condition, such as diabetes or hypertension, were not key predictors of willingness to share wearable data. Past studies of US adults have found these to be significant correlates of reported data sharing from electronic medical devices (eg, glucometers and blood pressure monitors) [19]. Therefore, along with prior research [20], the findings of this study demonstrate the importance of examining behavioral predictors and outcomes of patient-generated data sharing for different types of technology.

In this study, level of physical activity and health self-efficacy were significant health-related correlates of willingness to share wearable data with providers. Previous research has shown that wearable users tend to be more physically active than the general population [1]; however, the association between physical activity and willingness of wearable users to share data with providers has been unclear to date [2]. Therefore, our study results contribute to the literature that, among wearable users, those with higher versus lower levels of physical activity may be more willing to share their data with providers. Moreover, although research suggests that using a wearable device may increase health self-efficacy [21,35], our findings suggest that users with relatively high versus low health self-efficacy may be more willing to share their wearable data. Because wearable users with lower levels of physical activity or lower health self-efficacy may benefit the most from sharing wearable data with receptive providers (eg, individualized counseling), future mHealth intervention studies could include these factors in intervention design and explore how to overcome these potential barriers to data sharing. An increasingly acknowledged digital divide that has arisen from disparate health or behavioral outcomes among technology users [36] makes such research particularly important.

The results of this study also showed that trust in health information from providers is a strong predictor of willingness to share wearable data with them. Previous studies of US adults also found an association between trust in providers and willingness to exchange lifestyle behavior information via mHealth technologies, such as smartphones or tablets [18]. To increase willingness to share wearable data among those with lower levels of trust in providers as information sources, future mHealth intervention studies could explore ways to build trust in health information exchange within the patient–provider–technology relationship.

The second aim of our study was to investigate the relationship between digital health behaviors and willingness of users to share wearable data. The study findings showed that those who reported using their wearables every day or almost every day were more likely to report willingness to share data with providers than those who used them less often. Those who reported using (vs not using) smartphones or tablets to help communicate with providers were also more likely to report willingness to share. Consistent with prior research [21,22,25], these results suggest that greater use and technology self-efficacy, specifically in the context of health care and the patient–provider relationship, may increase the intention to share wearable health information with providers. These may be important targets for future intervention research focused on increasing health information exchange with providers via wearables.

By contrast, using SNSs to share health information and participating in a web-based health community were not significantly correlated with willingness to share health information from wearables with providers. These findings suggest that health information–sharing behaviors may vary based on the context (eg, health care setting or online support group), the audience or recipient (eg, health care providers or peers), and the technology through which the information is shared. Because SNSs and web-based health communities may be helpful to individuals through the visibility, availability, control, and reach they offer [37,38], future mHealth intervention studies that aim to improve wearable data sharing with providers could consider how to incorporate these factors into the intervention design.

#### Willingness to Share Wearable Data With Family or Friends

This study also aimed to explore the correlates of willingness to share data with family and friends, as there may be different drivers of willingness to share data based on the recipient of the information [2]. Controlling for other factors, including sociodemographics, health-related characteristics, and digital health behaviors (frequency of wearable use, use of SNSs to share health information, and participation in a web-based health community), only higher (vs lower) levels of physical activity were significantly associated with willingness to share wearable data with family and friends. As shown by Hyde et al [2], our findings suggest that there are distinctive drivers of intention to share health information via wearable health and activity trackers. However, we contribute to the literature the finding that physical activity of US adult wearable users appears to be a particularly important individual factor associated with willingness to share wearable health information given that physical activity was a strong predictor of willingness to share with both providers and family or friends.

One explanation for these findings is that individuals already engaged in health-promoting behaviors have higher health self-efficacy and are more willing to share their data because these data improve their ability to manage their health. Because social support and health self-efficacy are beneficial outcomes of sharing wearable health data with family or friends [9-11], individuals who may need support the most (those with low levels of physical activity) may be missing these benefits. To overcome barriers to sharing wearable data, such as lack of confidence in level of physical activity, mHealth interventions could be designed to work with participants in web-based health communities to focus on progress and on generating esteem support rather than focusing predominantly on social competition.

### Limitations

One of the limitations of this study is the reliance on self-reported and cross-sectional data. In addition, our study was limited by the inability to distinguish between various types of wearable health and activity trackers, which can vary considerably in their functionality. Due to limitations of the data set, we also could not assess other factors that potentially affect willingness to share wearable data with others, such as technology self-efficacy or concerns about privacy or data security.

### Conclusions

This study contributes to understanding the willingness of US adults to share data from wearable health and activity trackers with health care providers and family or friends. Several behavior-related factors were independently associated with willingness to share wearable data with providers, including level of physical activity, health self-efficacy, information-related trust in providers, frequency of wearable use, and use of mHealth technologies to help communicate with providers. Only level of physical activity was significantly associated with willingness to share wearable data with family or friends, controlling for other factors. Future behavioral surveillance research could assess attitudes associated with willingness to share wearable data, as well as factors that may influence these attitudes (eg, concerns about privacy), given the strong relationship between attitudes and behavioral intention [39]. In addition, given that attitudes about mHealth technologies and use of patient-generated data from wearables involve both patients and providers, researchers could use participatory action approaches that include these stakeholders in intervention design and implementation. When used as a communication tool, the potential of wearables to improve population health may not be fully realized without attention to these individual and relational factors.

## Appendix

Multimedia Appendix 1 Health Information National Trends Survey 5, Cycle 3 instrument.

Multimedia Appendix 2 Weighted, unadjusted population estimates for characteristics of the analytic sample of the Health Information National Trends Survey and that of wearable users.

## Abbreviations

HINTS: Health Information National Trends Survey
mHealth: mobile health
OR: odds ratio
SNS: social networking site
