# Supplementary material for: Willingness to Share Data From Wearable Health and Activity Trackers: Analysis of the 2019 Health Information National Trends Survey Data
Source: JMIR Mhealth Uhealth. 2021 Dec 13;9(12):e29190. doi: 10.2196/29190 (PMC8713093; doi:10.2196/29190)
Supplement: Multimedia Appendix 1 [file mhealth_v9i12e29190_app1.pdf]

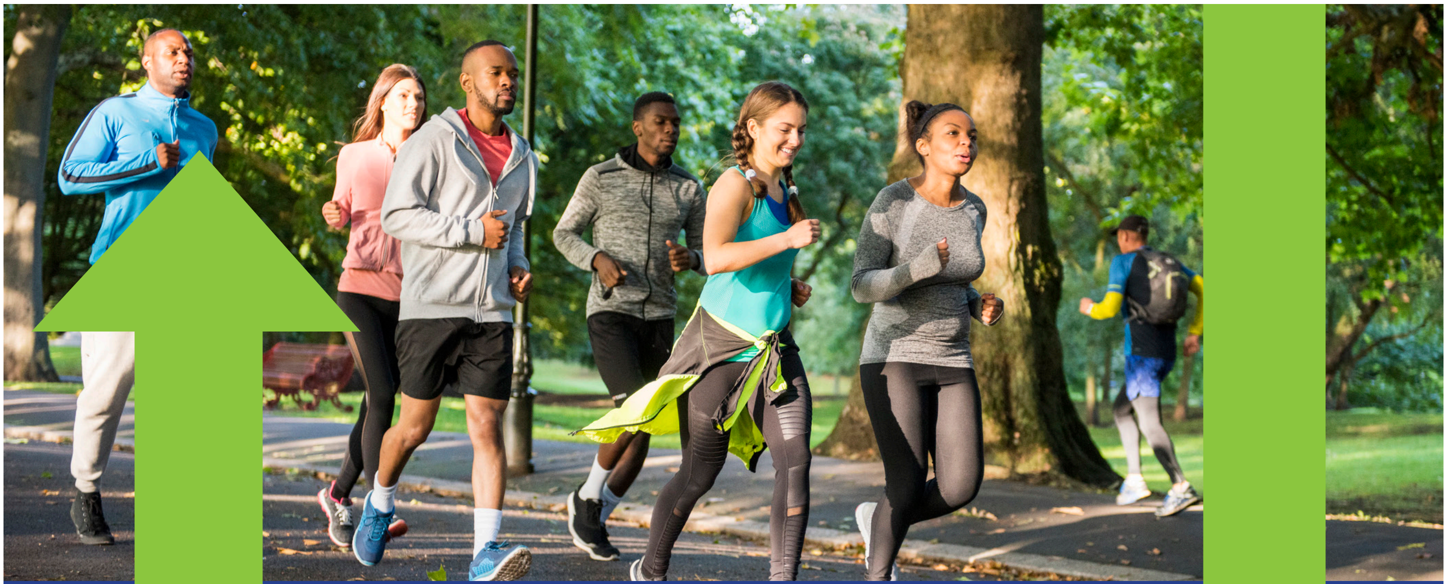

Health Information

National Trends Survey

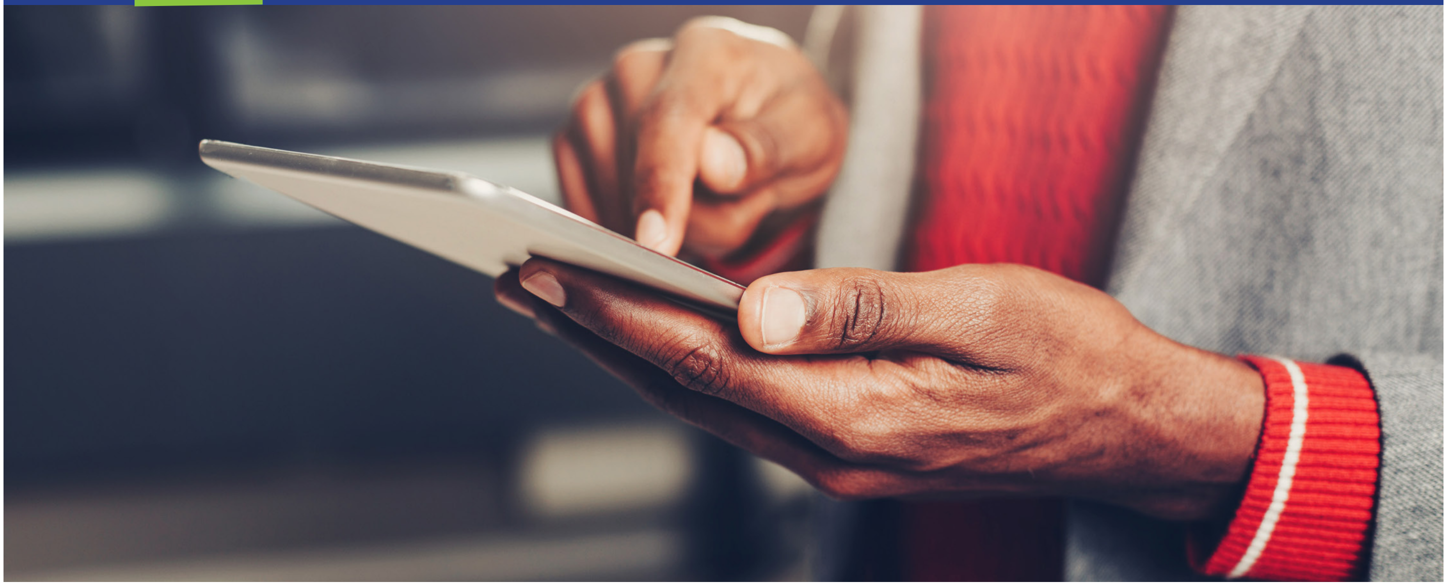

H5–C3

Annotated Form
Cycle 3/Web Pilot, English Version

START HERE:

Instructions

- Please use a black or blue pen to complete this form.
- Mark ☒ to indicate your answer.
- If you want to change your answer, mark ■ on the wrong answer.

1. Is there more than one person age 18 or older living in this household?

   AdultsInHH

   1 ☐ Yes

   2 ☐ No → **GO TO A1 on the next page**

2. Including yourself, how many people age 18 or older live in this household?

   MailHHAdults

   ☐☐

3. **The adult with the next birthday should complete this questionnaire.** This way, across all households, HINTS will include responses from adults of all ages.

4. Please write the first name, nickname, or initials of the adult with the next birthday. This is the person who should complete the questionnaire.

   ☐

Si prefiere recibir la encuesta en español, por favor llame 1-888-738-6812

36491

A: Looking For Health Information

A1. Have you ever looked for information about health or medical topics from any source?

SeekHealthInfo

1 Yes

2 No → **GO TO A5 in the next column**

A2. The most recent time you looked for information about health or medical topics, where did you go first?

Mark only one. WhereSeekHealthInfo WhereSeekHealthInfo_IMP

1 Books

2 Brochures, pamphlets, etc.

3 Cancer organization

4 Family

5 Friend/Co-worker

6 Doctor or health care provider

7 Internet

8 Library

9 Magazines

10 Newspapers

11 Telephone information number

12 Complementary, alternative, or unconventional practitioner

A3. The most recent time you looked for information about health or medical topics, who was it for?

WhoLookingFor

1 Myself

2 Someone else

3 Both myself and someone else

A4. Based on the results of your most recent search for information about health or medical topics, how much do you agree or disagree with each of the following statements?

	Strongly agree	Somewhat agree	Somewhat disagree	Strongly disagree
a. It took a lot of effort to get the information you needed............. LotofEffort	1	2	3	4
b. You felt frustrated during your search for the information......... Frustrated	1	2	3	4

A5. Overall, how confident are you that you could get advice or information about health or medical topics if you needed it?

ConfidentGetHealthInf

1 Completely confident

2 Very confident

3 Somewhat confident

4 A little confident

5 Not confident at all

A6. In general, how much would you trust information about health or medical topics from each of the following?

	Not at all	A little	Some	A lot
a. A doctor................................. TrustDoctor	4	3	2	1
b. Family or friends..................... TrustFamily	4	3	2	1
c. Government health agencies... TrustGov	4	3	2	1
d. Charitable organizations.......... TrustCharities	4	3	2	1
e. Religious organizations and leaders................................... TrustReligiousOrgs	4	3	2	1

36491

A7. Imagine that you had a strong need to get information about health or medical topics. Where would you go first?

Mark only <u>one</u>. StrongNeedHealthInfo StrongNeedHealthInfo_IMP

1 Books
2 Brochures, pamphlets, etc.
3 Cancer organization
4 Family
5 Friend/Co-worker
6 Doctor or health care provider
7 Internet
8 Library
9 Magazines
10 Newspapers
11 Telephone information number
12 Complementary, alternative, or unconventional practitioner
91 Other – Specify → StrongNeedHealthInfo_OS

A8. Have you ever looked for information about <u>cancer</u> from any source?
SeekCancerInfo
1 Yes
2 No

B: Using the Internet to Find Information

B1. Do you ever go on-line to access the Internet or World Wide Web, or to send and receive e-mail?
UseInternet
1 Yes
2 No → **GO TO B5 on the next page**

B2. When you use the Internet, do you access it through...

	Yes	No
a. A regular dial-up telephone line.................... Internet_DialUp	1	2
b. Broadband such as DSL, cable, or FiOS..... Internet_BroadBnd	1	2
c. A cellular network (i.e., phone, 3G/4G)........ Internet_Cell	1	2
d. A wireless network (Wi-Fi)........................... Internet_WiFi	1	2

B3. <u>In the past 12 months</u>, have you used the Internet to look for information about cancer for yourself?
InternetCancerInfoSelf
1 Yes
2 No

B4. How often do you access the Internet through each of the following?

	Daily	Sometimes	Never	Not Applicable
a. Computer at home..................... WhereUseInternet_Home	1	2	3	4
b. Computer at work..................... WhereUseInternet_Work	1	2	3	4
c. Computer in a public place (library, community center, other).. WhereUseInternet_PublicPlace	1	2	3	4
d. On a mobile device (cell phone/smart phone/tablet)........ WhereUseInternet_MobileDevice	1	2	3	4

36491

B5. In the past 12 months, have you used a computer, smartphone, or other electronic means to do any of the following?

	Yes	No
a. Looked for health or medical information for yourself................ Electronic_SelfHealthInfo	1	2
b. Bought medicine or vitamins online............ Electronic_BuyMedicine	1	2
c. Used e-mail or the Internet to communicate with a doctor or a doctor's office... Electronic_TalkDoctor	1	2
d. Tracked health care charges and costs..... Electronic_TrackedHealthCosts	1	2
e. Looked up medical test results.................. Electronic_TestResults	1	2
f. Made appointments with a health care provider.. Electronic_MadeAppts	1	2
g. Looked for information about the **harms** of electronic or e-cigarettes (also known as vapes, vape-pens, tanks, mods, or pod-mods)... Electronic_ECigHarms	1	2

B6. Please indicate if you have each of the following.

Mark all that apply.

1 Tablet computer (for example, an iPad, Samsung Galaxy, Motorola Xoom, or Kindle Fire) HaveDevice_Tablet

1 Smartphone (for example, an iPhone, Android, Blackberry, or Windows phone) HaveDevice_SmartPh

1 Basic cell phone only HaveDevice_CellPh

1 I do not have any of the above HaveDevice_None

GO TO B9 in the next column

HaveDevice_Cat

B7. On your tablet or smartphone, do you have any "apps" related to health and wellness?

TabletHealthWellnessApps

1 Yes

2 No

3 Don't know

B8. Has your tablet or smartphone…

	Yes	No
a. Helped you track progress on a health-related goal such as quitting smoking, losing weight, or increasing physical activity?...................................... Tablet_AchieveGoal	1	2
b. Helped you make a decision about how to treat an illness or condition?................. Tablet_MakeDecision	1	2
c. Helped you in discussions with your health care provider?............................... Tablet_DiscussionsHCP	1	2

B9. In the past 12 months, have you used an electronic wearable device to monitor or track your health or activity? For example, a Fitbit, Apple Watch, or Garmin Vivofit.

WearableDevTrackHealth

1 Yes

2 No → **GO TO B12 below**

B10. In the past month, how often did you use a wearable device to track your health?

FreqWearDevTrackHealth

1 Every day

2 Almost every day

3 1-2 times per week

4 Less than once per week

5 I did not use a wearable device in the past month

B11. Would you be willing to share health data from your wearable device with...

	Yes	No
a. your health care provider?.......................... WillingShareData_HCP	1	2
b. your family or friends?................................ WillingShareData_Fam	1	2

B12. In the last 12 months, have you used an electronic medical device to monitor or track your health? For example a glucometer or digital blood pressure device.

OtherDevTrackHealth2

1 Yes

2 No

36491

B13. Have you shared health information from either an electronic monitoring device or smartphone with a health professional within the last 12 months?

SharedHealthDeviceInfo

1 Yes

2 No

3 Not Applicable

B14. Sometimes people use the Internet to connect with other people online through social networks like Facebook or Twitter. This is often called “social media”.

In the past 12 months, have you used the Internet for any of the following reasons?

	Yes	No
a. To visit a social networking site, such as Facebook or LinkedIn................................. IntRsn_VisitedSocNet	1	2
b. To share health information on social networking sites, such as Facebook or Twitter... IntRsn_SharedSocNet	1	2
c. To write in an online diary or blog (i.e., Web log).. IntRsn_WroteBlog	1	2
d. To participate in an online forum or support group for people with a similar health or medical issue................................ IntRsn_SupportGroup	1	2
e. To watch a health-related video on YouTube... IntRsn_YouTube	1	2

B15. Have you sent a text message to or received a text message from a doctor or other health care professional within the last 12 months?

TextFromDoctor

1 Yes

2 No

3 Don't know

C: Your Health Care

C1. Not including psychiatrists and other mental health professionals, is there a particular doctor, nurse, or other health professional that you see most often?

RegularProvider

1 Yes

2 No

C2. In the past 12 months, not counting times you went to an emergency room, how many times did you go to a doctor, nurse, or other health professional to get care for yourself?

FreqGoProvider

0 None → **GO TO C4 on the next page**

1 1 time

2 2 times

3 3 times

4 4 times

5 5-9 times

6 10 or more times

C3. Overall, how would you rate the quality of health care you received in the past 12 months?

QualityCare

1 Excellent

2 Very good

3 Good

4 Fair

5 Poor

36491

C4. Urgent care, walk-in or retail clinics are healthcare providers that allow people to come in without an appointment. They do not include visits to the emergency room.

How many times in the past 12 months have you visited an urgent care, walk-in or retail clinic to get care for yourself?

FreqGoUrgentCare

0 I have not visited an urgent care, walk-in or retail clinic in the past 12 months → **SEE INSTRUCTIONS IN THE BOX BELOW**

1 1 time
2 2-4 times
3 5-9 times
4 10 or more times

C5. Overall, how would you rate the quality of health care you received from urgent care, walk-in or retail clinics in the past 12 months?

QualityCareUrgentCare

1 Excellent
2 Very good
3 Good
4 Fair
5 Poor

If you have not seen any health care professionals in the last 12 months then go to C7 in the next column.

Otherwise, go to C6 in the next column.

C6. The following questions are about your communication with all doctors, nurses, or other health professionals you saw during the past 12 months.

How often did they do each of the following?	Always	Usually	Sometimes	Never
a. Give you the chance to ask all the health-related questions you had............ ChanceAskQuestions	1	2	3	4
b. Give the attention you needed to your feelings and emotions........... FeelingsAddressed	1	2	3	4
c. Involve you in decisions about your health care as much as you wanted............ InvolvedDecisions	1	2	3	4
d. Make sure you understood the things you needed to do to take care of your health...................... UnderstoodNextSteps	1	2	3	4
e. Explain things in a way you could understand........................ ExplainedClearly	1	2	3	4
f. Spend enough time with you........ SpentEnoughTime	1	2	3	4
g. Help you deal with feelings of uncertainty about your health or health care.................................. HelpUncertainty	1	2	3	4

C7. Are you currently covered by any of the following types of health insurance or health coverage plans?

	Yes	No
a. Insurance through a current or former employer or union.................................. HealthIns_InsuranceEmp	1	2
b. Insurance purchased directly from an insurance company.................................. HealthIns_InsurancePriv	1	2
c. Medicare, for people 65 and older, or people with certain disabilities................ HealthIns_Medicare	1	2
d. Medicaid, Medical Assistance, or any kind of government-assistance plan for those with low incomes or a disability...... HealthIns_Medicaid	1	2
e. TRICARE or other military health care..... HealthIns_Tricare	1	2
f. VA (including those who have ever used or enrolled for VA health care)................. HealthIns_VA	1	2
g. Indian Health Service.............................. HealthIns_IHS	1	2
h. Any other type of health insurance or health coverage plan (Specify)................ HealthIns_Other	1	2

HealthIns_Other_OS

36491

D: Medical Records

Next, we are going to ask you some questions about your medical records. Medical records are defined as medical history, such as laboratory test results, clinical notes, and current list of medications.

D1. Do any of your doctors or other health care providers maintain your medical records in a computerized system?
ProviderMaintainEMR2
1 Yes
2 No
3 Don't Know

D2. Have you ever been offered online access to your medical records by your…

	Yes	No	Don't know
a. health care provider?........................... OfferedAccessHCP2	1	2	3
b. health insurer?.................................... OfferedAccessInsurer2	1	2	3

D3. How many times did you access your online medical record in the last 12 months?
AccessOnlineRecord
0 0
1 1 to 2 times
2 3 to 5 times
3 6 to 9 times
4 10 or more times

1 to 2 times, 3 to 5 times, 6 to 9 times, 10 or more times: **GO TO D5 on the next page**

D4. Why have you not accessed your medical record online? Is it because…

	Yes	No
a. You prefer to speak to your health care provider directly?.................................... NotAccessed_SpeakDirectly	1	2
b. You do not have a way to access the website?.. NotAccessed_NoInternet	1	2
c. You did not have a need to use your online medical record?............................ NotAccessed_NoNeed	1	2
d. You were concerned about the privacy or security of the website that had your medical records?.................................... NotAccessed_ConcernedPrivacy	1	2
e. You don't have an online medical record?.. NotAccessed_NoRecord	1	2
f. You found it difficult to login (for example, you had trouble remembering your password)?...................................... NotAccessed_LogInProb	1	2
g. You are not comfortable or experienced with computers?... NotAccessed_Uncomfortable	1	2
h. You have more than one online medical record?.. NotAccessed_MultipleRec	1	2

!

If you have not accessed any medical records in the last 12 months, go to Section E.

Otherwise, go to D5 on the next page.

36491

D5. In the past 12 months, have you used your online medical record to...

	Yes	No
a. Request refill of medications?...................... RecordsOnline_RefillMeds	1	2
b. Look up test results?.................................... RecordsOnline_ViewResults	1	2
c. Request correction of inaccurate information?.. RecordsOnline_RequestCorrection	1	2
d. Securely message health care provider and staff (for example, e-mail)?..................... RecordsOnline_MessageHCP	1	2
e. Download your health information to your computer or mobile device, such as a cell phone or tablet?... RecordsOnline_DownloadHealth	1	2
f. Add health information to share with your health care provider, such as health concerns, symptoms, and side effects?........ RecordsOnline_AddHealthInfo	1	2
g. Help you make a decision about how to treat an illness or condition?........................ RecordsOnline_MakeDecision	1	2

D6. Did you use a smartphone health app to access your online medical record?

AccessUsingHealthApp

1 Yes

2 No

3 Don't Know

D7. Do any of your online medical records include clinical notes (health provider's notes that describe a visit)?

OnlineRecClinNotes

1 Yes

2 No

3 Don't Know

D8. Have you electronically sent your medical information to…?

	Yes	No
a. Another health care provider?....................... ESent_AnotherHCP	1	2
b. A family member or another person involved with your care?... ESent_Family	1	2
c. A service or app that can help manage and store your health information?...................... ESent_HealthApp	1	2

D9. How easy or difficult was it to understand the health information in your online medical record?

UnderstandOnlineMedRec

1 Very easy

2 Somewhat easy

3 Somewhat difficult

4 Very difficult

D10. In general, how useful is your online medical record for monitoring your health?

UsefulOnlineMedRec

1 Very useful

2 Somewhat useful

3 Not very useful

4 Not at all useful

5 I do not use my online medical records to monitor my health

36491

E: Caregiving

E1. Are you currently caring for or making health care decisions for someone with a **medical, behavioral, disability, or other condition?**

Mark all that apply.

[1] Yes, a child/children
Caregiving_Child
[1] Yes, a spouse/partner
Caregiving_Spouse
[1] Yes, a parent/parents
Caregiving_Parent
[1] Yes, another family member
Caregiving_AnotherFam
[1] Yes, a friend or other non-relative
Caregiving_Friend
[1] No → **GO TO Section F on the next page**
Caregiving_No

CaregivingWho_Cat

E2. Do you provide any of this care professionally as part of a job (for example, as a nurse or professional home health aide)?

Caregiving_Professional
[1] Yes
[2] No

E3. Think about the individual for whom you are currently providing the most care. About how many hours per week do you spend in an average week providing care?

Caregiving_HoursPerWeek2
[| |] Hours spent providing care per week

E4. Please think about the individual for whom you are currently providing the most care.

Please check all conditions for which you have provided care for this person.

Mark all that apply.

[1] **Cancer**
Caregiving_Cancer
[1] **Alzheimer's, confusion, dementia, forgetfulness**
Caregiving_Alzheimers
[1] **Orthopedic/Musculoskeletal Issues** (examples: back problems, broken bones, arthritis, mobility problems, can't get around, feeble, unsteady, falling)
Caregiving_OrthoMusc
[1] **Mental health/behavioral/substance abuse issues** (examples: mental illness, emotional problems, depression, anxiety, substance/drug/alcohol abuse)
Caregiving_MentalHealth
[1] **Chronic conditions** (examples: high blood pressure/hypertension, diabetes, heart disease, heart attack, lung disease, emphysema, Chronic Obstructive Pulmonary Disease (COPD), Parkinson's)
Caregiving_ChronicCond
[1] **Neurological/developmental Issues** (examples: brain damage or injury, developmental or intellectual disorder, mental retardation, Down syndrome, stroke)
Caregiving_NeuroDev
[1] **Acute conditions**
Caregiving_AcuteCond
[1] **Aging/aging related health issues not listed in the other categories above**
Caregiving_Aging
[1] **Other – Specify** → Caregiving_Other Caregiving_Other_OS
[1] **Not sure/don't know**
Caregiving_NotSure

CaregivingCond_Cat

E5. Think about the individual for whom you are currently providing the most care. How many times did you access your care recipient's online medical record in the last 12 months?

Caregiving_AccessMedRec
[0] None
[1] 1 to 2 times
[2] 3 to 5 times
[3] 6 to 9 times
[4] 10 or more times

36491

F: Your Overall Health

F1. In general, would you say your health is...
GeneralHealth
1 Excellent,
2 Very good,
3 Good,
4 Fair, or
5 Poor?

F2. Overall, how confident are you about your ability to take good care of your health?
OwnAbilityTakeCareHealth
1 Completely confident
2 Very confident
3 Somewhat confident
4 A little confident
5 Not confident at all

F3. Some people avoid visiting their doctor even when they suspect they should. Would you say this is true for you, or not true for you?
AvoidDoc
1 True
2 Not true

F4. Are you deaf or do you have serious difficulty hearing?
Deaf
1 Yes
2 No

F5. Do you have friends or family members that you talk to about your health?
TalkHealthFriends
1 Yes
2 No

F6. Has a doctor or other health professional ever told you that you had any of the following medical conditions:

	Yes	No
a. Diabetes or high blood sugar?...................... MedConditions_Diabetes	1	2
b. High blood pressure or hypertension?.......... MedConditions_HighBP	1	2
c. A heart condition such as heart attack, angina, or congestive heart failure?............. MedConditions_HeartCondition	1	2
d. Chronic lung disease, asthma, emphysema, or chronic bronchitis?............. MedConditions_LungDisease	1	2
e. Depression or anxiety disorder?.................. MedConditions_Depression	1	2

F7. About how tall are you without shoes?

[ ] Feet ***and*** [ ][ ] Inches
Height_Feet; Height_Inches

F8. About how much do you weigh, in pounds, without shoes?

[ ][ ][ ] Weight
Pounds

F9. Right now, do you feel you are...
WeightPerception
1 Overweight,
2 Slightly overweight,
3 Underweight,
4 Slightly underweight, or
5 Just about the right weight for you?

F10. At any time in the past year, have you intentionally tried to…
WeightIntention
1 Lose weight,
2 Maintain your weight,
3 Gain weight, or
4 You haven't really paid attention to your weight?

36491

F11. Over the past 2 weeks, how often have you been bothered by any of the following problems?

	Nearly every day	More than half the days	Several days	Not at all
a. Little interest or pleasure in doing things........................... LittleInterest	1	2	3	4
b. Feeling down, depressed, or hopeless................................. Hopeless	1	2	3	4
c. Feeling nervous, anxious, or on edge................................. Nervous	1	2	3	4
d. Not being able to stop or control worrying...................... Worrying	1	2	3	4

F12. To what extent do you agree or disagree with the following statements?

	Strongly agree	Somewhat agree	Somewhat disagree	Strongly disagree
a. I control my emotions by changing the way I am thinking about the situation I'm in.............. ChangeThinking	1	2	3	4
b. I consider how things might be in the future, and try to influence those things with my day to day behavior .... ConsiderFuture	1	2	3	4

G: Health and Nutrition

G1. About how many cups of fruit (including 100% pure fruit juice) do you eat or drink each day?

Fruit
- 0 None
- 1 ½ cup or less
- 2 ½ cup to 1 cup
- 3 1 to 2 cups
- 4 2 to 3 cups
- 5 3 to 4 cups
- 6 4 or more cups

1 cup of fruit could be:
- 1 small apple
- 1 large banana
- 1 large orange
- 8 large strawberries
- 1 medium pear
- 2 large plums
- 32 seedless grapes
- 1 cup (8 oz.) fruit juice
- ½ cup dried fruit
- 1 inch-thick wedge of watermelon

G2. About how many cups of vegetables (including 100% pure vegetable juice) do you eat or drink each day?

Vegetables
- 0 None
- 1 ½ cup or less
- 2 ½ cup to 1 cup
- 3 1 to 2 cups
- 4 2 to 3 cups
- 5 3 to 4 cups
- 6 4 or more cups

1 cup of vegetables could be:
- 3 broccoli spears
- 1 cup cooked leafy greens
- 2 cups lettuce or raw greens
- 12 baby carrots
- 1 medium potato
- 1 large sweet potato
- 1 large ear of corn
- 1 large raw tomato
- 2 large celery sticks
- 1 cup of cooked beans

G3. About how many calories do you think a man/woman of your age and physical activity needs to consume a day to maintain your current weight?

[ ][ ][ ][ ] AverageCaloriesPerDay Calories

AverageCaloriesPerDay_DK
- 8 Don't know

G4. Think about the last time you ordered food in a fast food or sit down restaurant, did you notice calorie information listed next to the food on the menu or menu board?

NoticeCalorieInfoOnMenu

1 Yes

2 No → **GO TO G7 in the next column**

G5. Thinking about the last time you noticed calorie information on the menu or menu board, how easy or difficult to understand was the calorie information?

UnderstandCalorieInfo

1 Very easy

2 Somewhat easy

3 Somewhat difficult

4 Very difficult

G6. Thinking about the last time you noticed calorie information on the menu or menu board, how did the calorie information change what you were thinking of ordering?

	Yes	No
a. I ordered something with fewer calories...... CalorieInfo_FewerCalories	1	2
b. I ordered something with more calories....... CalorieInfo_MoreCalories	1	2
c. I ordered fewer items.................................. CalorieInfo_FewerItems	1	2
d. I ordered smaller sizes................................ CalorieInfo_SmallerSizes	1	2
e. I ordered more items.................................. CalorieInfo_MoreItems	1	2
f. I ordered larger sizes.................................. CalorieInfo_LargerSizes	1	2

G7. These are examples of one drink of alcohol:

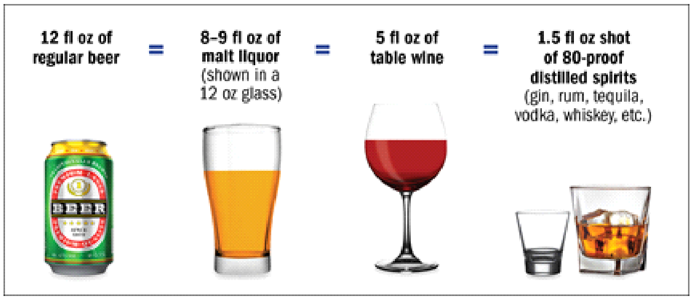

During the past 30 days, how many days per week did you have at least one drink of any alcoholic beverage?

DrinkDaysPerWeek

☐ Days per week

(IF 0 THEN GO TO G9 BELOW)

G8. During the past 30 days, on the days when you drank, about how many drinks did you drink on average?

DrinksPerDay

☐☐ Average drinks per day

G9. Which of the following health conditions do you think can result from drinking too much alcohol?

	Yes	No	Don't know
a. Cancer.. AlcoholConditions_Cancer	1	2	3
b. Heart Disease.................................... AlcoholConditions_HeartDisease	1	2	3
c. Diabetes... AlcoholConditions_Diabetes	1	2	3
d. Liver disease....................................... AlcoholConditions_LiverDisease	1	2	3

G10. In the past 12 months, how much have you heard about the negative health consequences of drinking alcohol from a doctor or other health care professional?

HCPAlcoholConsequences

1 A lot

2 Some

3 A little

4 Nothing

5 I have not seen a doctor or health professional in the past 12 months

36491

H: Physical Activity and Exercise

H1. In a typical week, how many days do you do any physical activity or exercise of at least moderate intensity, such as brisk walking, bicycling at a regular pace, and swimming at a regular pace (do not include weightlifting)?

TimesModerateExercise

- 0 None → **GO TO H3 below**
- 1 1 day per week
- 2 2 days per week
- 3 3 days per week
- 4 4 days per week
- 5 5 days per week
- 6 6 days per week
- 7 7 days per week

H2. On the days that you do any physical activity or exercise of at least moderate intensity, how long do you typically do these activities?

HowLongModerateExerciseMinutes

☐☐☐ **Minutes per day**

H3. In a typical week, outside of your job or work around the house, how many days do you do leisure-time physical activities specifically designed to strengthen your muscles such as lifting weights or circuit training (do not include cardio exercise such as walking, biking, or swimming)?

TimesStrengthTraining

- 0 None
- 1 1 day per week
- 2 2 days per week
- 3 3 days per week
- 4 4 days per week
- 5 5 days per week
- 6 6 days per week
- 7 7 days per week

H4. During the past 7 days, how much time did you spend sitting on a typical day at home or at work? This may include time spent sitting at a desk, visiting friends, reading, driving or riding in a car, or sitting or lying down to watch television.

AverageTimeSitting

☐☐ **Hours per day**

H5. To what extent do you enjoy exercising?

EnjoyExercise

- 4 Not at all
- 3 A little
- 2 Some
- 1 A lot

H6. People start or continue exercising regularly for lots of reasons. How much do each of the following reflect why you would start or continue exercising regularly?

	Not at all	A little	Some	A lot
a. Pressure from others................. RegExercise_Pressure	4	3	2	1
b. Concern over the way you look.. RegExercise_Appearance	4	3	2	1
c. Feeling guilty when you skip exercising.................................. RegExercise_Guilt	4	3	2	1
d. Getting enjoyment from exercise.................................... RegExercise_Enjoyment	4	3	2	1

36491

H7. The Federal Government publishes the Physical Activity Guidelines for Americans, which provide recommendations for how much physical activity to get to be healthy. In the past 6 months, have you heard about government recommendations for physical activity from any of the following sources?

	Yes	No
a. Health professional or doctor........................ GovPARec_HCP	1	2
b. Social media or Internet................................ GovPARec_Internet	1	2
c. Television.. GovPARec_TV	1	2
d. Magazine... GovPARec_Magazine	1	2

H8. Think about the last time you heard a new government recommendation about physical activity or exercise. Which of the following best describe what you did in response to the new recommendation?

Mark all that apply.

1 I increased the amount of physical activity/exercise that I do
ExRec_IncreasedEx
1 I decreased the amount of physical activity/exercise that I do
ExRec_DecreasedEx
1 I changed the type of physical activity that I do
ExRec_ChangedEx
1 I looked for more information about the recommendation
ExRec_LookedInfo
1 I did not change what I do
ExRec_NoChange
1 I have not heard any government recommendations about physical activity or exercise
ExRec_NotHeard

ExRec_Cat

H9. As far as you know, does physical activity…

	Yes	No	Don't know
a. Help with sleep?............................... PhysAct_HelpSleep	1	2	3
b. Reduce anxiety and depression?..... PhysAct_ReduceAnxiety	1	2	3
c. Reduce pain?.................................... PhysAct_ReducePain	1	2	3

H10. During the past 7 days, how many hours of sleep did you get on average per night?

AverageSleepNight
☐☐ **Hours of sleep per night**

H11. In the past 7 days, how would you rate your sleep quality overall?
AverageSleepQuality
1 Very good
2 Fairly good
3 Fairly bad
4 Very bad

H12. Someone might describe themselves as a "morning-person" or "night-person." Which do you consider yourself to be?
MorningNightPerson
1 I'm definitely a morning-person
2 I'm more of a morning-person than a night-person
3 I'm neither a morning-person nor a night-person
4 I'm more of a night-person than a morning-person
5 I'm definitely a night-person

36491

J: Sun & UV Exposure

J1. On warm sunny days, how often do you spend time in the sun in order to get a tan?

SpendTimeInSunTanning

- 1 Often
- 2 Sometimes
- 3 Rarely
- 4 Never
- 5 Don't go out on sunny days

J2. To what extent do you enjoy spending time in the sun?

EnjoyTimeInSun

- 4 Not at all
- 3 A little
- 2 Some
- 1 A lot

J3. During the past 12 months, how many times have you had a sunburn (even a small part of your skin turns red or hurts for 12 hours or more) from too much sun exposure?

[| |] TimesSunburned

Sunburns in past 12 months

(IF 0 THEN GO TO SECTION K ON THE NEXT PAGE)

J4. On the most recent time you were sunburned, what were you doing when you were sunburned?

Mark <u>all that apply</u>.

- 1 Working at your job
  Sunburned_JobOutside
- 1 Working outside at your own home or a family/friend's home
  Sunburned_HomeOutside
- 1 Sunbathing
  Sunburned_Sunbathing
- 1 Swimming
  Sunburned_Swimming
- 1 Exercise (running, hiking, sports) (do not include swimming)
  Sunburned_Exercise
- 1 Watching a sporting event
  Sunburned_SportingEvent
- 1 Attending an outdoor event or venue (a concert, the zoo, a fair, etc.)
  Sunburned_OutdoorEvent
- 1 Day-to-day activities
  Sunburned_DayToDay
- 1 Other
  Sunburned_Other
- 1 Don't know
  Sunburned_DK

SunburnedAct_Cat

J5. The most recent time you got sunburned, were you doing any of the following things to protect yourself from the sun?

Mark <u>all that apply</u>.

- 1 Wearing sunscreen with SPF of at least 15
  Sunburned_SPF15
- 1 Wearing protective clothing such as long pants or a shirt with sleeves that cover your shoulders
  Sunburned_ProtClothing
- 1 Staying in the shade or under an umbrella
  Sunburned_Shade
- 1 None of the above
  Sunburned_None
- 1 I don't know/I don't remember
  Sunburned_DontRemember

SunburnedProt_Cat

J6. Were you drinking alcohol at any of the times when you were sunburned?

Sunburned_Alcohol

- 1 Yes
- 2 No

36491

K: Tobacco Products

K1. Have you smoked at least 100 cigarettes in your entire life?

Smoke100

1 Yes

2 No → **GO TO K5 below**

K2. How often do you now smoke cigarettes?

SmokeNow

1 Every day

2 Some days

3 Not at all → **GO TO K5 below**

K3. At any time in the past year, have you stopped smoking for one day or longer because you were trying to quit?

TriedQuit

1 Yes

2 No

K4. Are you seriously considering quitting smoking in the next six months?

ConsiderQuit

1 Yes

2 No

K5. New types of cigarettes are now available called electronic cigarettes or e-cigarettes (also known as vapes, vape-pens, tanks, mods or pod-mods). These products deliver nicotine through a vapor. Compared to smoking cigarettes, would you say that electronic cigarettes are…

ElectCigLessHarm

1 Much less harmful,

2 Less harmful,

3 Just as harmful,

4 More harmful,

5 Much more harmful, or

7 I don't know

K6. Have you ever used an e-cigarette, even one or two times?

UsedECigEver

1 Yes

2 No → **GO TO K9 on the next page**

K7. Do you now use an e-cigarette every day, some days, or not at all?

UseECigNow

1 Every day

2 Some days

3 Not at all

K8. During the past 30 days, on how many days did you use e-cigarettes?

SmokeDayECig

0 0 days

1 1 or 2 days

2 3 to 5 days

3 6 to 9 days

4 10 to 19 days

5 20 to 29 days

6 All 30 days

K9. How much do you agree or disagree with the following statements?

	Strongly agree	Agree	Disagree	Strongly disagree	Don't know
a. Nicotine is the main substance in tobacco that makes people want to smoke............................ NicotineWantSmoke	1	2	3	4	5
b. The nicotine in cigarettes is the substance that causes most of the cancer caused by smoking................................ NicotineCauseCancer	1	2	3	4	5
c. Addiction to nicotine is something that I am concerned about....................... NicotineAddictionConcern	1	2	3	4	5

K10. Compared to a typical cigarette, would you think that a cigarette advertised as "low nicotine" would be…

LowNicotineHarmful

- 5 Much less harmful to your health than a typical cigarette?
- 4 Slightly less harmful to your health than a typical cigarette?
- 3 Equally harmful to your health as a typical cigarette?
- 2 Slightly more harmful to your health than a typical cigarette?
- 1 Much more harmful to your health than a typical cigarette?

K11. Compared to a typical cigarette, would you think that a cigarette advertised as "low nicotine" would be…

LowNicotineAddictive

- 5 Much less addictive than a typical cigarette?
- 4 Slightly less addictive than a typical cigarette?
- 3 Equally addictive as a typical cigarette?
- 2 Slightly more addictive than a typical cigarette?
- 1 Much more addictive than a typical cigarette?

K12. In the past 12 months, have you seen messages saying that a Federal Court has ordered tobacco companies to make statements about the dangers of smoking cigarettes? These messages have been in newspapers, on television, on tobacco company websites, and on cigarette packs.

SeenFederalcourtTobaccoMessages2

- 1 Yes
- 2 No → **GO TO L1 on the next page**

K13. Which of the following messages have you seen?

Mark all that apply.

- 1 That a Federal Court has ordered tobacco companies to make statements about the health effects of smoking. TobaccoMessages_HESmoking
- 1 That a Federal Court has ordered tobacco companies to make statements about the health effects of secondhand smoke. TobaccoMessages_HESecondhand
- 1 That a Federal Court has ordered tobacco companies to make statements about the addictiveness of smoking and nicotine. TobaccoMessages_Addictiveness
- 1 That a Federal Court has ordered tobacco companies to make statements about how cigarettes are designed to enhance the delivery of nicotine. TobaccoMessages_EnhanceDelivery
- 1 That a Federal Court has ordered tobacco companies to make statements about low tar and light cigarettes being just as harmful as regular cigarettes. TobaccoMessages_LowTarLight

TobaccoMessages_Cat

36491

L: Cancer Screening and Awareness

L1. Are you male or female?

GenderC

1 Male

2 Female → **GO TO L3 below**

L2. A PSA test is used to check for prostate cancer. Have you ever had a PSA test?

EverHadPSATest

1 Yes

2 No

} **Males GO TO L5 in the next column**

L3. How long ago did you have your most recent Pap test to check for cervical cancer?

WhenPapTest

1 A year ago or less

2 More than 1, up to 2 years ago

3 More than 2, up to 3 years ago

4 More than 3, up to 5 years ago

5 More than 5 years ago

6 I have never had a Pap test

L4. When did you have your most recent mammogram to check for breast cancer, if ever?

WhenMammogram

1 A year ago or less

2 More than 1, up to 2 years ago

3 More than 2, up to 3 years ago

4 More than 3, up to 5 years ago

5 More than 5 years ago

6 I have never had a mammogram

L5. There are a few different tests to check for colon cancer. These tests include:

A **colonoscopy** – For this test, a tube is inserted into your rectum and you are given medication that may make you feel sleepy. After the procedure, you need someone to drive you home.

A **sigmoidoscopy** – For this test, you are awake when the tube is inserted into your rectum. After the test you can drive yourself home.

A **stool blood test** – For this test, you collect a stool sample at home, and then provide it to a doctor or lab for testing

Have you ever had one of these tests to check for colon cancer?

EverTestedColonCa

1 Yes

2 No

L6. Have you ever heard of the Hepatitis C virus (also known as Hep C or **HCV**)?

HeardHepC

1 Yes

2 No

L7. Have you ever heard of **HPV**? HPV stands for Human Papillomavirus. It is not HCV, HIV, HSV, or herpes.

HeardHPV

1 Yes

2 No → **GO TO L9 below**

L8. Do you think **HPV** can cause…

	**Yes**	**No**	**Not sure**
a. Cervical Cancer?............................... HPVCauseCancer_Cervical	1	2	3
b. Penile Cancer?................................. HPVCauseCancer_Penile	1	2	3
c. Anal Cancer?................................... HPVCauseCancer_Anal	1	2	3
d. Oral Cancer?................................... HPVCauseCancer_Oral	1	2	3

L9. A vaccine to prevent **HPV** infection is available and is called the HPV shot, cervical cancer vaccine, GARDASIL®.

Before today, have you ever heard of the cervical cancer vaccine or HPV shot?

HeardHPVVaccine2

1 Yes

2 No

36491

M: Your Cancer History

M1. Have you ever been diagnosed as having cancer?

EverHadCancer

1 Yes

2 No → **GO TO N1 in the next column**

M2. What type of cancer did you have?

Mark all that apply.

1 Bladder cancer CaBladder
1 Bone cancer CaBone
1 Breast cancer CaBreast
1 Cervical cancer (cancer of the cervix) CaCervical
1 Colon cancer CaColon
1 Endometrial cancer (cancer of the uterus) CaEndometrial
1 Head and neck cancer CaHeadNeck
1 Leukemia/Blood cancer CaLeukemia
1 Liver cancer CaLiver
1 Lung cancer CaLung
1 Lymphoma (Hodgkin's) CaHodgkins
1 Lymphoma (Non-Hodgkin's) CaNonHodgkin
1 Melanoma CaMelanoma
1 Oral cancer CaOral
1 Ovarian cancer CaOvarian
1 Pancreatic cancer CaPancreatic
1 Pharyngeal (throat) cancer CaPharyngeal
1 Prostate cancer CaProstate
1 Rectal cancer CaRectal
1 Renal (kidney) cancer CaRenal
1 Skin cancer, non-melanoma CaSkin
1 Stomach cancer CaStomach
1 Other – Specify → CaOther CaOther_OS

Cancer_Cat

M3. At what age were you first told that you had cancer?

WhenDiagnosedCancer

☐☐☐ Age

GO TO N3 in the next column

N: Beliefs About Cancer

Think about cancer in general when answering the questions in this section.

N1. How likely are you to get cancer in your lifetime?

ChanceGetCancer

1 Very unlikely

2 Unlikely

3 Neither unlikely nor likely

4 Likely

5 Very likely

N2. How worried are you about getting cancer?

FreqWorryCancer

1 Not at all

2 Slightly

3 Somewhat

4 Moderately

5 Extremely

N3. Have any of your family members ever had cancer?

FamilyEverHadCancer

1 Yes

2 No

4 Not sure

36491

N4. How much do you agree or disagree with each of the following statements?

	Strongly agree	Somewhat agree	Somewhat disagree	Strongly disagree
a. It seems like everything causes cancer.. EverythingCauseCancer	1	2	3	4
b. There's not much you can do to lower your chances of getting cancer.. PreventNotPossible	1	2	3	4
c. There are so many different recommendations about preventing cancer, it's hard to know which ones to follow........... TooManyRecommendations	1	2	3	4

N5. Do you think the following could be a sign of cancer?

	Yes	No	Don't know
a. Unexplained bleeding........................ CancerSign_UnexpBleeding	1	2	3
b. A change in bowel or bladder habits.. CancerSign_BowelBladderChange	1	2	3
c. Unexplained weight loss................... CancerSign_UnexpWeightLoss	1	2	3

N6. How much do you think that each of the following can influence whether or not a person will develop cancer?

	A lot	A little	Not at all	Don't know
a. Being overweight or obese........ InfluenceCancer_Obesity	1	2	3	4
b. Eating enough fiber.................... InfluenceCancer_EatingFiber	1	2	3	4
c. Eating too much processed meat.. InfluenceCancer_ProcessedMeat	1	2	3	4
d. Eating fruits and vegetables ..... InfluenceCancer_EatingFruitVeg	1	2	3	4

O: You and Your Household

O1. What is your age?

Age

☐☐☐ Years old

O2. What is your marital status?

Mark only <u>one</u>.

MaritalStatus

1 Married
2 Living as married or living with a romantic partner
3 Divorced
4 Widowed
5 Separated
6 Single, never been married

O3. What is the highest grade or level of schooling you completed?

Education

1 Less than 8 years
2 8 through 11 years
3 12 years or completed high school
4 Post high school training other than college (vocational or technical)
5 Some college
6 College graduate
7 Postgraduate

O4. How well do you speak English?

SpeakEnglish

1 Very well
2 Well
3 Not well
4 Not at all

36491

O5. Are you of Hispanic, Latino/a, or Spanish origin? One or more categories may be selected.

Mark all that apply.

- 1 No, not of Hispanic, Latino/a, or Spanish origin NotHisp
- 1 Yes, Mexican, Mexican American, Chicano/a Mexican
- 1 Yes, Puerto Rican PuertoRican
- 1 Yes, Cuban Cuban
- 1 Yes, another Hispanic, Latino/a, or Spanish origin OthHisp

Hisp_Cat

O6. What is your race? One or more categories may be selected.

Mark all that apply.

- 1 White White
- 1 Black or African American Black
- 1 American Indian or Alaska Native AmerInd
- 1 Asian Indian AsInd
- 1 Chinese Chinese
- 1 Filipino Filipino
- 1 Japanese Japanese
- 1 Korean Korean
- 1 Vietnamese Vietnamese
- 1 Other Asian OthAsian
- 1 Native Hawaiian Hawaiian
- 1 Guamanian or Chamorro Guamanian
- 1 Samoan Samoan
- 1 Other Pacific Islander OthPacIsl

Race_Cat2

O7. Do you think of yourself as…

SexualOrientation

- 1 Heterosexual, or straight
- 2 Homosexual, or gay or lesbian
- 3 Bisexual
- 91 Something else – Specify

SexualOrientation_OS

SexualOrientation_I

O8. Including yourself, how many people live in your household?

TotalHousehold
Number of people

O9. Starting with yourself, please mark the sex, and write in the age and month of birth for each adult 18 years of age or older living at this address.

	Sex	Age	Month Born (01-12)
**SELF**	1 Male 2 Female SelfGender	SelfAge	SelfMOB
Adult 2	1 Male 2 Female HHAdultGender2	HHAdultAge2	HHAdultMOB2
Adult 3	1 Male 2 Female HHAdultGender3	HHAdultAge3	HHAdultMOB3
Adult 4	1 Male 2 Female HHAdultGender4	HHAdultAge4	HHAdultMOB4
Adult 5	1 Male 2 Female HHAdultGender5	HHAdultAge5	HHAdultMOB5

O10. How many children under the age of 18 live in your household?

ChildrenInHH
Number of children under 18

O11. Do you currently rent or own your home?

RentOrOwn

- 1 Own
- 2 Rent
- 3 Occupied without paying monetary rent

36491

O12. Thinking about members of your family living in this household, what is your combined annual income, meaning the total pre-tax income from all sources earned in the past year?

IncomeRanges

- 1 $0 to $9,999
- 2 $10,000 to $14,999
- 3 $15,000 to $19,999
- 4 $20,000 to $34,999
- 5 $35,000 to $49,999
- 6 $50,000 to $74,999
- 7 $75,000 to $99,999
- 8 $100,000 to $199,999
- 9 $200,000 or more

O13. Which one of these comes closest to your own feelings about your household's income?

IncomeFeelings

- 1 Living comfortably on present income
- 2 Getting by on present income
- 3 Finding it difficult on present income
- 4 Finding it very difficult on present income

Thank you!

Please return this questionnaire in the postage-paid envelope within 2 weeks.

If you have lost the envelope, mail the completed questionnaire to:

HINTS Study, TC 1046F
Westat
1600 Research Boulevard
Rockville, MD 20850

36491
