# Supplementary material for: Willingness to Share Data From Wearable Health and Activity Trackers: Analysis of the 2019 Health Information National Trends Survey Data
Source: JMIR Mhealth Uhealth. 2021 Dec 13;9(12):e29190. doi: 10.2196/29190 (PMC8713093; doi:10.2196/29190)
Supplement: Multimedia Appendix 2 [file mhealth_v9i12e29190_app2.docx]

Multimedia Appendix Table 2. Weighted, unadjusted population estimates for characteristics of the HINTS^a^ analytic sample and analytic sample of wearable users

| Characteristic | Analytic sample (N=5,438)  n (weighted %, SE) | Wearable users (n=1,300)  n (weighted %, SE) |
| --- | --- | --- |
| **Sex, n (%)** | N/A^b^ | N/A |
| Men | 2,236 (48.80, 0.19) | 486 (44.97, 2.08) |
| Women | 3,073 (51.20, 0.19) | 787 (55.03, 2.08) |
| **Age, years, n (%)** | N/A | N/A |
| 18-34 | 687 (24.27, 0.89) | 272 (33.62, 2.08) |
| 35-49 | 968 (24.48, 0.98) | 339 (30.48, 1.85) |
| 50-64 | 1,668 (31.10, 0.83) | 411 (26.37, 1.88) |
| ≥ 65 | 1,961 (20.15, 0.09) | 257 (9.53, 0.81) |
| **Race and ethnicity, n (%)** | N/A | N/A |
| White, non-Hispanic | 3,054 (63.47, 0.30) | 788 (64.69, 1.83) |
| Black, non-Hispanic | 677 (11.31, 0.21) | 141 (8.86, 1.19) |
| Hispanic | 730 (16.82, 0.14) | 169 (17.66, 1.64) |
| Other race or ethnicity | 389 (8.39, 0.24) | 106 (8.79, 1.18) |
| **Education, n (%)** | N/A | N/A |
| College graduate or postgraduate | 1,280 (30.37, 0.66) | 796 (42.55, 1.97) |
| Technical, vocational, or some college | 1,591 (40.16, 0.65) | 344 (42.45, 2.38) |
| High school graduate or less | 2,410 (29.47, 0.15) | 130 (15.00, 2.07) |
| **Annual household income, U.S. $, n (%)** | N/A | N/A |
| ≥ $100,000 | 1,325 (25.66, 1.08) | 526 (38.26, 2.25) |
| $75,000-$99,999 | 643 (12.86, 0.69) | 211 (17.72, 1.56) |
| $50,000-$74,999 | 905 (16.82, 0.81) | 219 (16.18, 1.58) |
| $35,000-$49,999 | 735 (14.11, 0.76) | 142 (13.80, 1.81) |
| < 35,000 | 1,757 (30.55, 1.11) | 192 (14.04, 1.77) |
| **Geographic area, n (%)** | N/A | N/A |
| Urban | 4,837 (86.74, 0.70) | 1,196 (88.07, 1.91) |
| Rural | 601 (13.26, 0.70) | 104 (11.93, 1.91) |
| **Perceived health status, n (%)** | N/A | N/A |
| Poor or fair | 853 (15.16, 0.81) | 113 (10.35, 1.65) |
| Good | 4,484 (84.84, 0.81) | 1,174 (89.65, 1.65) |
| **Health self-efficacy, n (%)** | N/A | N/A |
| Lower | 1,486 (28.45, 1.08) | 278 (22.85, 1.97) |
| Higher | 3,861 (71.55, 1.08) | 1,006 (77.15, 1.97) |
| **Regular health care provider, n (%)** | N/A | N/A |
| No | 1,609 (35.51, 1.19) | 379 (35.27, 2.28) |
| Yes | 3,724 (64.49, 1.19) | 904 (64.73, 2.28) |
| **Trust health information from doctor, n (%)** | N/A | N/A |
| Lower | 1,570 (31.04, 1.12)) | 321 (24.45, 1.90) |
| Higher | 3,725 (68.96, 1.12) | 955 (75.55, 1.90) |
| **Trust health information from family or friends, n (%)** | N/A | N/A |
| Lower | 4,701 (90.94, 0.77) | 1167 (90.89, 1.64) |
| Higher | 405 (9.06, 0.77) | 89 (9.11, 1.64) |
| **BMI, n (%)** | N/A | N/A |
| 18.5-24.9 (normal) | 1,583 (30.53, 1.04) | 389 (30.56, 1.92) |
| 25-29.9 (overweight) | 1,826 (35.36, 1.18) | 472 (38.87, 2.44) |
| ≥ 30 (obese) | 1,770 (34.11, 1.21) | 395 (30.57, 2.15) |
| **Multimorbidity, n (%)** | N/A | N/A |
| 0 conditions | 1,758 (40.98, 1.11) | 545 (46.29, 2.30) |
| 1 condition | 1,654 (31.45, 1.11) | 398 (31.56, 2.31) |
| 2+ conditions | 1,798 (27.57, 1.01) | 326 (22.15, 2.02) |
| **Level of physical activity, n (%)** | N/A | N/A |
| Lower | 3,371 (62.04, 1.12) | 674 (51.46, 2.18) |
| Higher | 1,881 (37.96, 1.12) | 595 (48.54, 2.18) |
| **Use of mhealth technologies to help communicate with providers, n (%)** | N/A | N/A |
| No | 2,716 (63.03, 1.10) | 635 (52.07, 2.18) |
| Yes | 1,728 (36.97, 1.10) | 617 (47.93, 2.18) |
| **Use social networking sites to share health information, n (%)** | N/A | N/A |
| No | 4,707 (85.38, 0.86) | 1,056 (80.46, 1.75) |
| Yes | 636 (14.62, 0.86) | 229 (19.54, 1.75) |
| **Participating in an online health community, n (%)** | N/A | N/A |
| No | 4,973 (91.89, 0.67) | 1,143 (88.05, 1.58) |
| Yes | 377 (8.11, 0.67) | 145 (11.95, 1.58) |

^a^HINTS 2019: Health Information National Trends Survey (HINTS) 5, Cycle 3

^b^N/A: Not applicable
